# Assessing organic material on single-use vessel sealing devices: a comparative study of reprocessed and new LigaSure™ devices

**DOI:** 10.1007/s00464-020-07969-8

**Published:** 2020-09-09

**Authors:** Swathi Ramesh Chivukula, Steven Lammers, Jennifer Wagner

**Affiliations:** Department of Bioengineering, University of Colorado Denver | Anschutz Medical Campus, 12705 E. Montview Blvd., Ste. 100, Mail Stop 8607, Aurora, CO 80045 USA

**Keywords:** Reprocessing, Devices, Design, Cleaning, Sterilization, Contamination

## Abstract

**Background:**

Reprocessed devices must be thoroughly cleaned prior to sterilization to ensure efficacy of sterilization agents. Many single-use devices are not designed to be thoroughly cleaned. Interlocking design features inherent to LigaSure™ vessel sealing devices may prevent thorough cleaning and promote accumulation of human tissue that cannot be removed. Thus, the aim of this study was to compare industry reprocessed and new LigaSure™ vessel sealing devices for organic material.

**Methods:**

A total of 168, 84 new and 84 reprocessed, vessel sealing devices were disassembled and inspected for the presence of residual organic matter using visual, microscopic, and chemical analysis. Devices were randomized and test conductors blinded to group membership. Devices were aseptically disassembled and sent through visual inspection. Next, devices were either examined using light microscopy, scanning electron microscopy (SEM) or exposed to a solution that luminesces in the presence of hemoglobin. Additionally, 165 reprocessed devices were sent to a 3rd party lab for sterility testing via direct immersion culture for 14 days.

**Results:**

Significant amounts of remnant organic material (C, N, O, S, Na, P) were observed with 81/84 reprocessed and 0/84 new devices failing inspection protocols. When tested for the presence of hemoglobin, only 1/12 reprocessed devices passed inspection. SEM of reprocessed devices revealed residues with liquid patterns and diffuse soiling with foreign material. Sterility testing of reprocessed devices revealed a sterility level < 6^–3^.

**Conclusions:**

The abundance of material resembling human tissue observed on reprocessed VSDs suggests inadequate cleaning prior to sterilization. Atomic and morphological analyses of the remnant materials suggest that bacterial biofilms could also be present. Additionally, surface degradation and release of reinforcing glass fibers from the device were observed. Devices designed for single use can harbor significant amounts of remnant material that likely interfere with the sterilization process.

Hospital-acquired infections place a significant burden on our medical system and patient outcomes with an estimated 1.7 million patients affected annually [1]. Approximately 1 in 17 of those infected die making hospital-acquired infections one of the top 10 leading causes of death in the United States [2]. The estimated annual financial burden to our medical system is $9.8 billion dollars, with surgical site infections accounting for approximately 34% of these costs [3].

One potential source of infection is surgical instrumentation used in the operating suite. Improperly sterilized instruments can act as fomites, transmitting infections to patients. While many surgical tools may be cleaned, sterilized, and re-used, many laparoscopic tools are designed as single-use devices (SUDs) that are packaged sterile and designed to be discarded following the procedure. Given that SUDs contribute significantly to waste streams and procedural costs, reprocessing of SUDs has been used as a cost-saving measure since the 1970s. Institutions began using 3^rd^ party vendors for reprocessing in the 1990s [4]. In 1999, the Food and Drug Administration (FDA) began working to regulate reprocessing operations, formalizing regulations in 2002 with the Medical Device User Fee Act [5]. There is a significant financial incentive to reprocess medical devices with some estimates reporting a 50% cost savings [6]. When analyzing reuse of SUDs in ambulatory surgery centers, an annual cost savings of $25,000 per operating room was calculated [7]. Despite lower purchase prices, the reprocessed SUDs may have quality issues that impact surgery, as previously noted [8], or create potential health risks, such as infection or inflammatory responses [4, 9], that could impact quality of life and add cost to procedures in unanticipated ways. Existing functional and safety evidence for reprocessed SUD devices have been reviewed [10]; however, we are not aware of studies that examined reprocessed bipolar vessel sealing devices.

The aim of this study was to evaluate 3rd party commercially reprocessed bipolar vessel sealing devices (VSDs) labeled as single use for the presence of remnant biological materials and compare them to their new-from-manufacturer counterparts. Market research using a commercially available database (IQVIA, Inc.) indicates that new LigaSure™ devices purchased directly from Medtronic comprise approximately 84% of the market share of vessel sealing devices, making them a good candidate for this study. It was hypothesized that, due to the complex geometry and interlocking features present on the VSDs, remnant biological material would accumulate on the device and potentially interfere with sterilization of reprocessed devices completed by a 3rd party manufacturer.

## Methods and procedures

### Study design

This observational study was designed to compare new LigaSure™ VSDs (Covidien; Mansfield, MA) with reprocessed LigaSure™ VSDs (Stryker Sustainability Solutions; Tempe, AZ). Sample size was constrained by reprocessed device availability. A total of 84 reprocessed devices were procured. An equal number of new devices were then provided by the original equipment manufacturer. It should be noted that reprocessed devices were inherently older than new devices as they had been cycled through an unknown number of uses. This resulted in a generational discrepancy between new and reprocessed devices. Device geometry was identical but new devices had a non-stick coating.

Two types of devices were assessed: open VSDs (LigaSure Impact™), for use in open procedures, and laparoscopic (LigaSure™ blunt tip) VSDs, for laparoscopic use, as shown in Table 1.

**Table 1:** Breakdown of devices used in study

	Covidien™—new devices	Stryker—reprocessed devices
Device type I	LigaSure™ Blunt Tip (LF1837, Laparoscopic)	LigaSure™ Blunt Tip (LF1637, Laparoscopic)
Device type II	LigaSure Impact™ (LF4418, Open)	LigaSure Impact™ (LF4318, Open)

The study required careful disassembly of the instruments while preventing cross-contamination so a detailed protocol was developed. The protocol focused on preventing exposure of workers to soiled devices, prevention of cross-contamination through tools or work surfaces, contamination from test conductors, and imaging parameter optimization. All test personnel were trained on the protocols and blinded to the device group by obscuring labels.

**Fig. 1:**
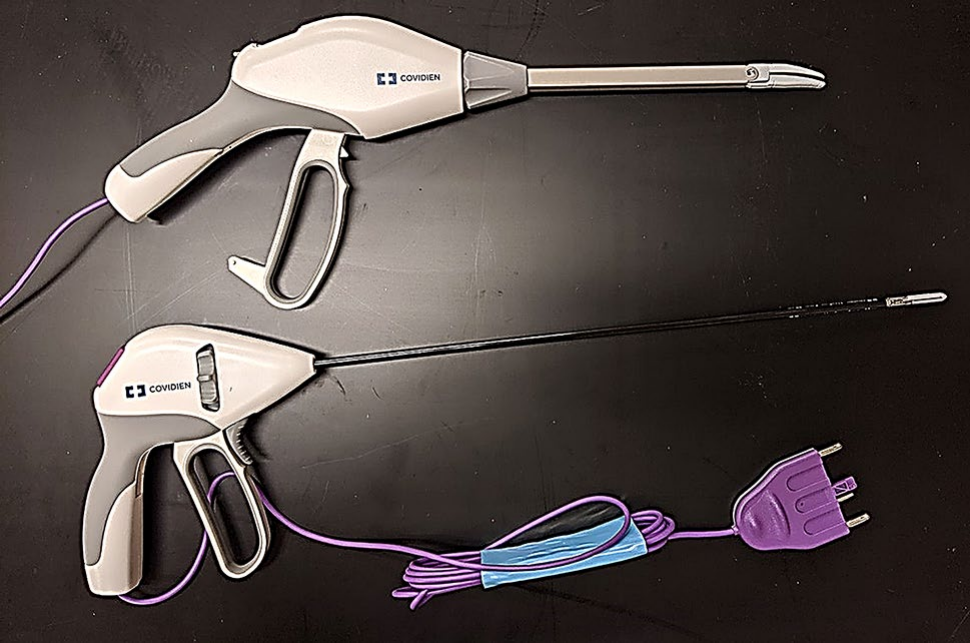
Devices under test. Upper image is of a new LigaSure Impact™ (open) device and lower image is of a new LigaSure™ Blunt Tip-(laparoscopic) device. Reprocessed devices had identical geometry but were from an older generation of devices

**Fig. 2:**
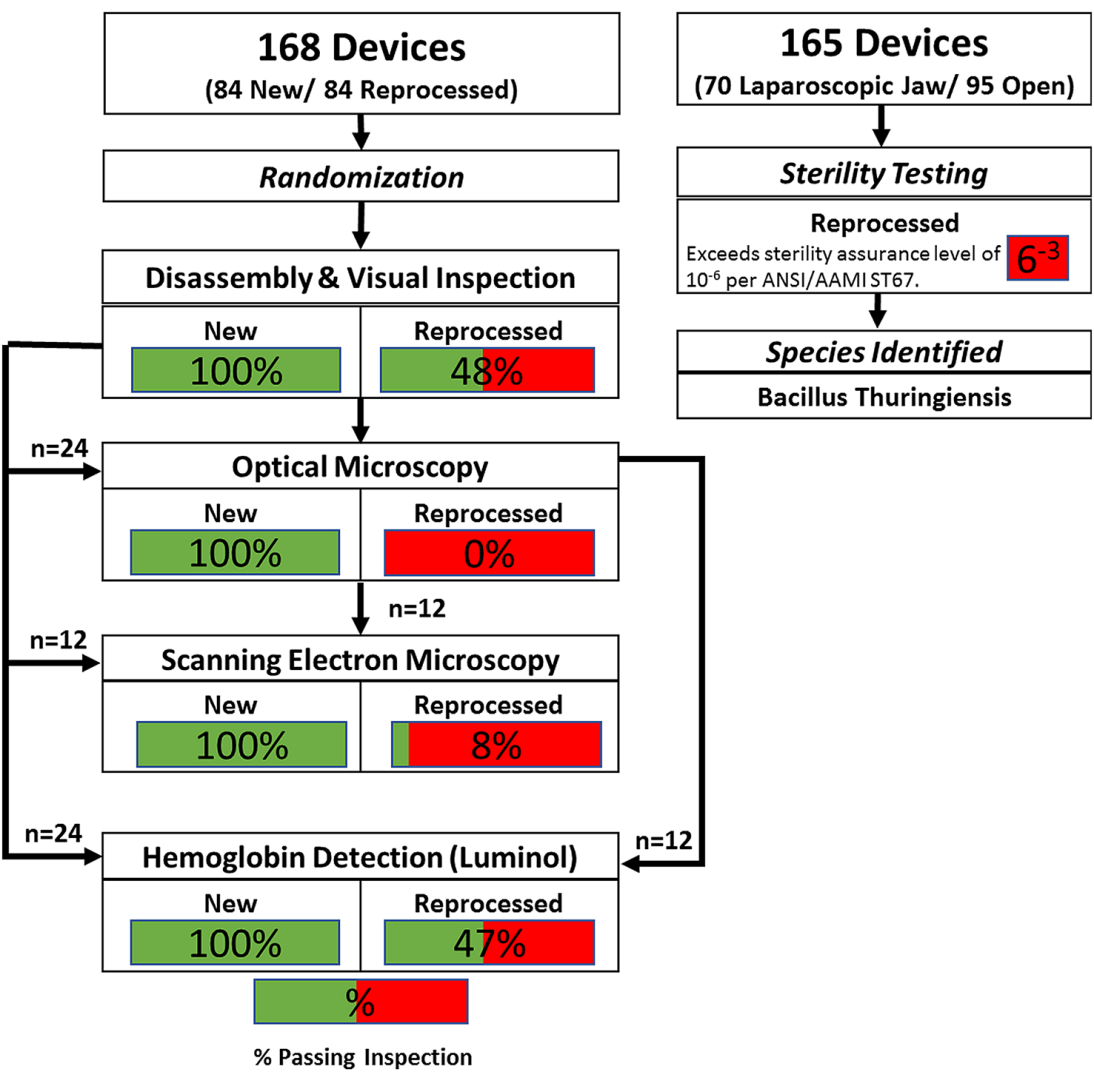
Flow chart of study design. Of the 168 devices tested (84 new and 84 reprocessed), devices were randomly assigned to groups as shown. Sterility testing was completed on a different set of 165 reprocessed devices

Devices are pictured in Fig. 1. A total of 168 devices were disassembled and studied with 84 new and 84 reprocessed instruments from each device family. All packaging and devices underwent visual inspection after which they were grouped and randomized for analytical testing including optical microscopy, scanning electron microscopy (SEM), EDS, and presumptive testing for hemoglobin (Fig. 2). Additionally, 165 instruments (70 laparoscopic, 95 open) were sent to a contract lab (Nelson Labs, Salt Lake City, UT) for sterility testing using direct immersion culture.

### Randomization, disassembly, and visual inspection procedures

First, device packaging was inspected for any breach of the sterile barrier. Devices were then grouped for subsequent testing prior to disassembly. During the disassembly process, devices were de-identified by placing tape over the location of labels on both new and reprocessed devices. Each device was then assigned an identification number. Personal protective equipment (PPE) worn by test conductors included lab coats, bouffant caps, safety glasses, face masks, and gloves. All tools and work surfaces were cleaned with soapy water and then disinfected with 70% ethanol. Clean foil was used as a barrier between all surfaces. During jaw disassembly, sterile gloves were used. At all times, caution was exercised to not make contact, either via instrument or glove, with regions of interest. Work areas were cleaned with ethanol and covered with new foil between each device to prevent cross-contamination.

Disassembled jaw, blade, and handle pieces were placed in sterile secondary containers marked only with their identification number. Disassembly resulted in 2 jaw pieces from each instrument, only one of which was tested. The remaining jaw pieces were stored in sterile containers for use as back-ups in the event of any testing issues. Throughout the disassembly process, all parts and pieces of the devices were examined for foreign material. Any foreign material observed was photographed and documented as a visual inspection failure.

### Optical and scanning electron microscopy

All regions of interest were observed using a 250 × digital USB microscope. Photographs of all regions at 2 MP resolution were obtained. Any foreign object matter or discoloration was documented. Next, samples were prepared for SEM imaging on the Children’s Hospital Colorado SEM system (JEOL JSM-6010LA, Peabody, MA, USA). Samples were first fixed in a 3:1 methanol:acetic acid solution and then transported to the imaging facility where they were sputter coated with gold and palladium. Images of all regions of interest were acquired and provided to the authors for analysis.

To characterize the morphology and atomic composition of the observed remnant material, EDS and higher magnification SEM imaging were performed on two samples, one new and one reprocessed, using the same instrumentation. First, areas of soiling on reprocessed devices were identified and a single sample was re-imaged at a higher magnification. For EDS analysis, one reprocessed device with visible soiling was selected for analysis along with a clean device identified as new. Only laparoscopic devices were used for supplemental analysis because the abundance of thick, red, visible soiling facilitated locating and testing remnant material. EDS analysis produces a spectrum of the atomic content on the surface, a table describing the mass percent for each identified element and a map of the location of each identified surface element. It should be noted that EDS is a surface characterization technique with a depth of characterization less than 500 nm.

### Hemoglobin detection

Luminol (Sigma Aldrich 123072) was mixed with an activated basic solution of Sodium Perborate Tetrahydrate (Sigma Aldrich 244120) and Sodium Carbonate Bioxtra (Sigma Aldrich S7795) according to manufacturer instructions. Devices were sprayed with a fine mist of solution and photographed using long exposures. A Canon Rebel Ti1 EFS with an 18–55 mm lens on a tripod was used to obtain images, in a dark room, with an aperture setting of 6.3, ISO speed of 100, and exposure time of 30 s. Reference images were also obtained prior to application of Luminol solution.

Copper, which is known to react with Luminol solutions, is present as a conductor to provide electricity to the seal plate. The wire was expected to luminesce, if not oxidized, so any copper wire that luminesced was considered an expected finding. Photographs were viewed on a Dell E228WFP monitor. Images with unexpected luminescence were considered positive for this presumptive hemoglobin test.

### Sterility testing

After visualizing thick, red soiling on numerous reprocessed devices, a total of 165 reprocessed LigaSure™ devices were sent for sterility testing at an independent facility (Nelson Labs, Salt Lake City, UT). Device jaws were aseptically cut down to size using isopropyl and flame sterilized bolt cutters to fit into media jars. In accordance with good manufacturing practices (GMP) and the International Standards Organization for Standardization standard 11737 (ISO 11737), devices were cultured for 14 days directly immersed in 2000 to 3000 mL of either soybean-casein digest broth (SCDB) at 20–25 °C or thioglycollate broth (THIO) at 30–35 °C. Cultures were then assessed for growth. Any growth was subsequently identified using morphologic and genetic analysis.

## Results

In total, 96% of reprocessed devices (81/84) and 0% of new devices (0/84) failed either visual inspection, microscopic inspection, or hemoglobin testing. Observation under SEM revealed structural damage to the instruments and the presence of what appears to be biological material. Details for each test segment are discussed below and results are summarized in Fig. 2.

### Visual inspection

**Fig. 3:**
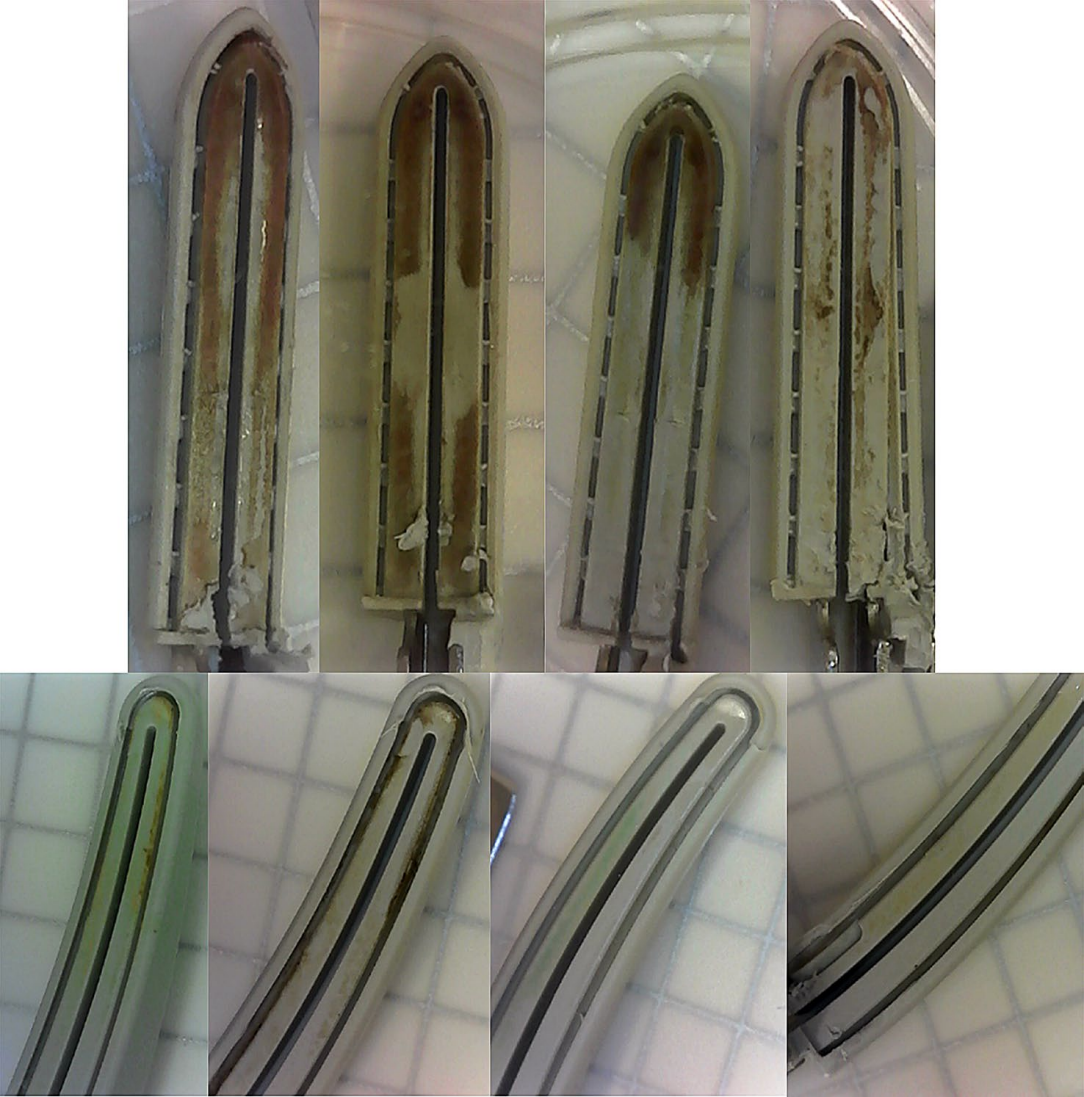
Photos of reprocessed devices with visual evidence of contamination. Top panel are reprocessed LigaSure(TM) Blunt Tip (LF1637, Stryker). Bottom panel are reprocessed LigaSure Impact(TM) (LF4318, Stryker)

All devices were opened from intact packaging and disassembled prior to assessing with the naked eye. Visual inspection of the disassembled devices resulted in 100% (84 of 84) of new devices and 48% (40 of 84) of reprocessed devices passing with an appearance devoid of foreign materials. Of note, all visible soiling was located in areas that cannot be visualized without complete device disassembly. The soiling on each reprocessed device type appeared uniquely. Soiling on the open devices consisted of diffuse red, yellow, brown, and/or green films, while laparoscopic devices presented with thick reddish-brown deposits (Fig. 3).

### Optical microscopy

Optical microscopy was performed on a portion of devices to detect soiling not visible to the naked eye. Six devices from each group were randomly chosen following visual inspection to undergo optical microscopy (Fig. 2). Consistent with visualization, 100% of new devices (12/12) passed inspection with optical microscopy with no evidence of foreign material. Of the reprocessed devices inspected with optical microscopy, 0% of devices passed inspection with all (12/12) having evidence of unexpected material present. Optical microscopy revealed remnant material not detected through visual inspection on two open devices.

### Scanning electron microscopy

**Fig. 4:**
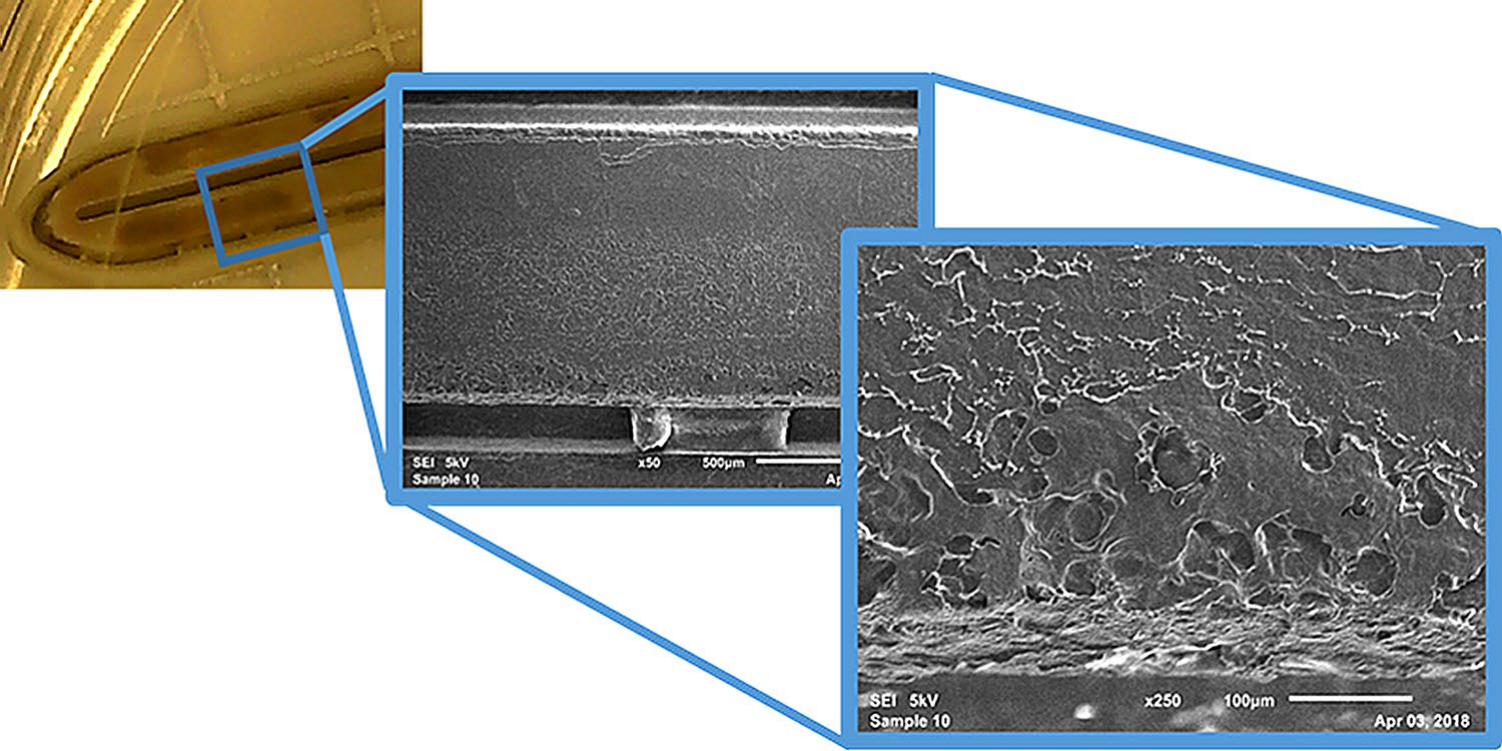
Example contamination on reprocessed device as shown by scanning electron microscopy. Example of surface from one LigaSure(TM) Blunt Tip (LF1637, Stryker) with visual surface contamination expanded to 50× (middle panel) and 250× (right panel)

**Fig. 5:**
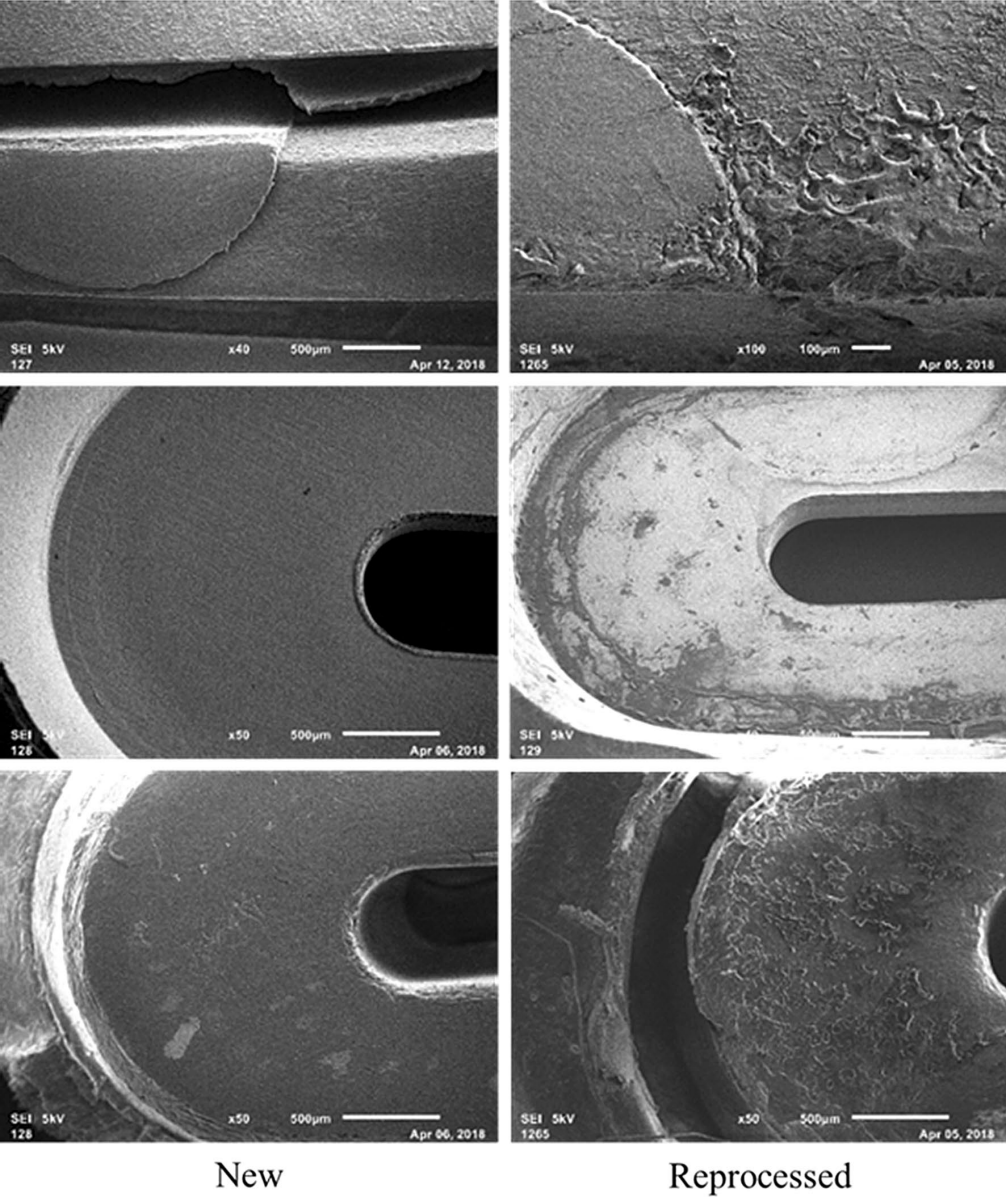
Representative images with scanning electron microscopy shows surface changes on reprocessed devices compared with new. The underside to the seal plates are shown in the middle row while the plastic jaws are pictured in the top and bottom rows. All photos displayed at 50×

Scanning Electron Microscopy (SEM) was employed on disassembled devices to reveal potential liquid residue that is indicative of biological soiling. Stark differences between new and reprocessed devices were observed. All new devices (12/12) passed inspection with no evidence of liquid residue (Fig. 2). Conversely, all but 1 reprocessed device (11/12) had thin and thick films which indicated liquid residue, indicative of biological soiling, as evidenced by the deposition pattern shown in Fig. 4. Thick films had deposition patterns consistent with that shown in Fig. 4 which shows the deposition pattern with increasing magnification. Figure 5 compares reprocessed open devices (left) with new open devices (right). The images shown are of the underside of the seal plate and the tip of the plastic jaw and are representative of the sample populations. Contrast differences between the metal plate (brighter) and the soil observed on the plate (darker) imply that the soil has a lower density than the metal plate, consistent with the expected density of biological materials.

**Fig. 6:**
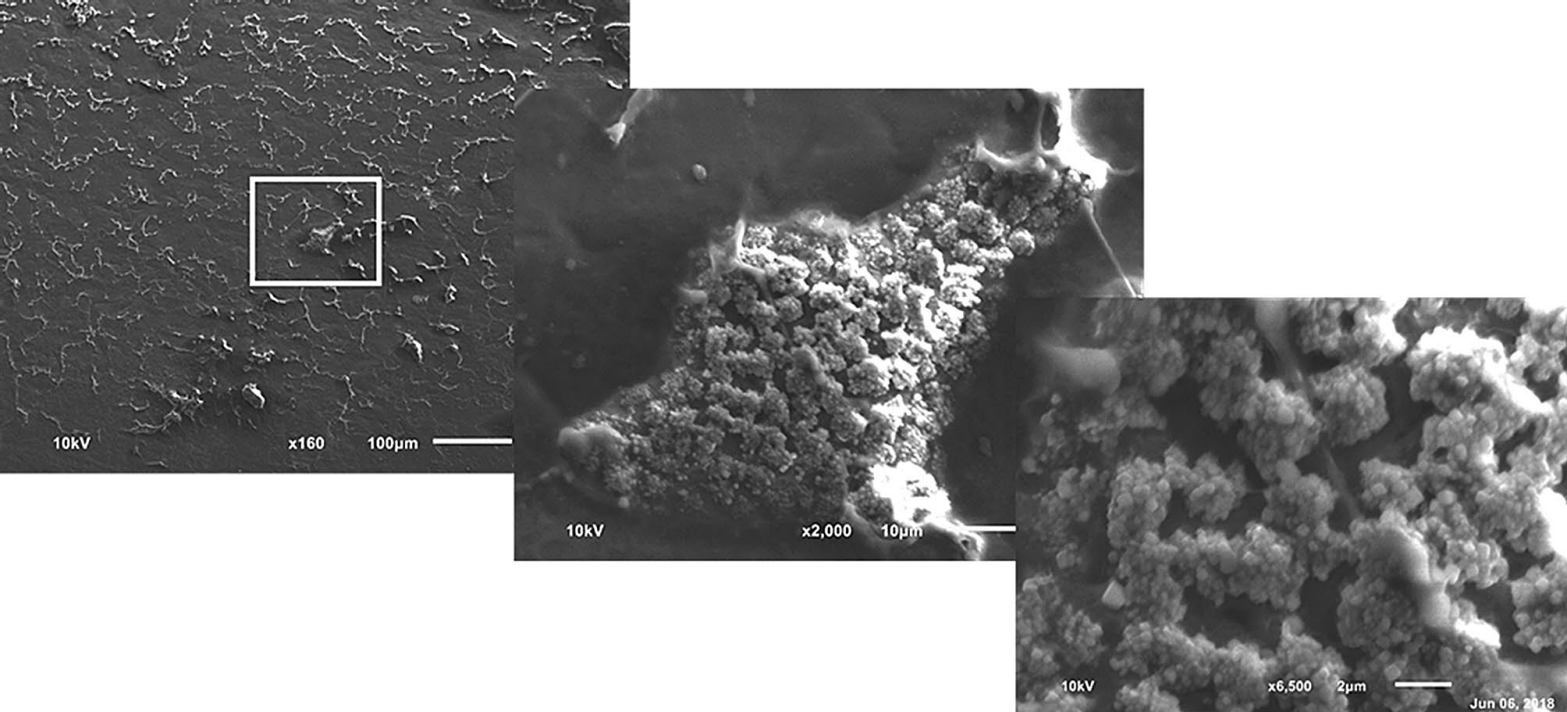
An area of contamination on a reprocessed device reveals structures resembling bacterial cocci. Scanning electron microscopy taken at 160× (right), 2000× (center), and 6500× (right)

Magnification was increased on an area with dense visible soiling to better characterize the thick residual material found on laparoscopic SUDs. Suspicious clusters that appear consistent in morphology with biofilms and/or bacterial colonies were observed throughout the remnant material. Clusters of spherical features between 0.5 μm and 1 μm in diameter, consistent in shape, size, and arrangement to cocci colonies, were observed and are shown in Fig. 6.

**Fig. 7:**
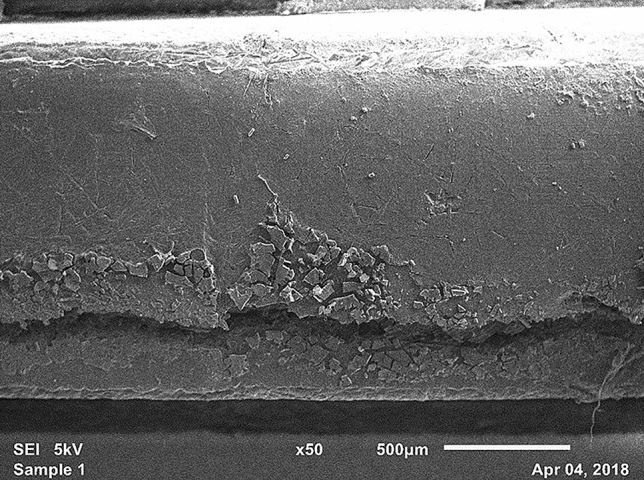
Surface degradation and glass fiber release observed using SEM on the plastic jaw of a reprocessed laparoscopic device. Scanning electron microscopy taken at 50×

Degradation of the polymeric structure was observed in reprocessed laparoscopic devices. Specifically, glass fibers used to reinforce the polymer were observed leaking out of the matrix and spreading diffusely about the device surfaces (Fig. 7). Also of note was the observation that reprocessed devices tended to undergo brittle failure during the disassembly process with the development of several large cracks as shown in Fig. 7.

### Energy dispersive X-ray spectroscopy (EDS)

The surfaces of the reprocessed device had a very different atomic composition when compared to the new device (Table 2). Analysis of the new device revealed the presence of primarily silicon, chromium, titanium, and oxygen reflecting the presence of glass fibers (silicon dioxide), titanium dioxide (protective surface coating), and a chromium oxide layer, thought to be transferred via mechanical contact between the stainless steel seal plate to the plastic jaw. The reprocessed device with residual material contained large amounts of carbon and nitrogen with small amounts of sodium, sulfur, and phosphorus, consistent with biological material [11]. Variability, mostly in phosphorus and sodium content, was observed across different areas of soiling. In general, phosphorus is present in biological materials as part of the phosphorylation process and in the DNA backbone. Sulfur is an element typically found in the presence of anaerobic bacteria and as components of the amino acids cysteine and methionine [12].

**Table 2:** Atomic composition of device surfaces

Energy dispersive X-ray spectroscopy results					
Clean area New device			Area with visible soiling Reprocessed device		
Chemical formula	Mass %	Atomic %	Chemical formula	Mass %	Atomic %
–	–	–	C	63.97	69.88
–	–	–	N	12.45	11.67
O	53.93	77.39	O	21.36	17.52
–	–	–	Na	0.34	0.20
Si	4.19	3.43	Si	0.09	0.04
–	–	–	P	0.65	0.28
–	–	–	S	0.80	0.33
Ti	18.28	8.76	Ti	0.33	0.09
Cr	23.59	10.42	–	–	–

### Hemoglobin detection

All new devices passed with negative luminescence detection, while only 47% of reprocessed devices (17/36) passed (Fig. 2). Luminol testing was positive for one reprocessed laparoscopic device that passed visual inspection. Devices with thick films did not luminesce uniformly but tended to glow around the periphery of the film. Luminescence was also observed in 3 reprocessed handle bodies (2 open devices, 1 laparoscopic device) indicating that biological materials can be deposited in all areas of the device.

### Sterility testing

Sterility testing was conducted on 165 reprocessed devices by a third party. One of the 165 samples cultured 1 exhibited growth (6^–3^) which is much lower than the sterility assurance level of 10^–6^ accepted by the FDA as laid out in the Association for the Advancement of Medical Instrumentation (AAMI) standards ST67 and TIR 12 for devices contacting normally sterile tissue. Growth observed during testing was identified as bacillus thuringiensis using gram stain, morphological and genetic analysis. Bacillus thuringiensis is a naturally occurring bacteria found in the soil.

## Discussion

Value-based healthcare is an important focus in the medical marketplace, and reprocessed medical devices, including SUDs, have emerged as a potential cost savings for medical facilities. The guidance and regulation of reprocessing SUDs has been developing over the past two decades; however, there remains a scarcity of data surrounding the effectiveness of reprocessing procedures. The benefits of reprocessing SUDs must be weighed against potential risks. The aim of this study was to evaluate the surfaces of bipolar vessel sealing devices (VSDs) labeled as SUDs for remnant biological materials by comparing 3rd party commercially reprocessed devices to the new-from-manufacturer counterparts. Overall, the assessments performed in this study showed that 96% of reprocessed devices (81/84) and 0% of new devices (0/84) failed either visual inspection, microscopic inspection, or hemoglobin testing.

The potential benefits of reprocessed SUDs are clear which has led to their growing use. Firstly, they may provide significant up-front cost saving to healthcare facilities when compared to new devices. New LigaSure™ devices retail for an average of $475 for a laparoscopic blunt tip device and $630 for an open Impact™ device (IQVIA, Inc.). Reprocessed versions of the same product retail for approximately $330 and $450, respectively, for an average cost savings of 30% ($138) per device. Another potential benefit is the environmental impact: collecting, reprocessing, and reusing SUDs can realize significant reductions in waste streams [10].

Conversely, the potential risks of reprocessing SUDs have not been robustly studied in practice. The success of reprocessing varies by device due to design complexity and the ability to properly clean a device prior to sterilization. In 2000, the FDA issued a draft guidance document surrounding the reprocessing of SUDs that states: “Some design features, such as narrow lumens and interlocking parts, can harbor debris that cannot be readily accessed and removed during cleaning” and that “if a device cannot be adequately cleaned, terminal processing to disinfect or sterilize the device will not be successful and the single-use device presents a greater risk of disease transmission [13].” The guidance document also classifies medical devices into three categories of risk: moderate, low and high, based upon both risk of infection and degradation leading to inadequate performance. Vessel sealing devices are classified in the high-risk category [13]. Along with surgical site infection, reprocessing risks also include pyrogenic reactions, particulate contamination, difficulty cleaning, toxicity, loss of manufacturer liability, and catastrophic device failure [4, 9]. As such, strict requirements for reprocessing critical devices are defined by the Joint Commission International. To minimize the risk of infection, all published guidelines mandate devices be thoroughly cleaned prior to disinfection and sterilization to ensure efficacy [4, 14–16].

Thorough cleaning prior to sterilization is necessary because remnant organic material has been shown to protect microbes from sterilization agents and lead to device-related infections [15], which likely occurs through physical and chemical interference with sterilization agents [17–22]. The United States Food and Drug Administration (FDA) issued a more recent guidance document for reprocessing medical devices that states all devices should be visually inspected and any devices that are visibly soiled should be either thoroughly cleaned prior to sterilization or safely disposed of [14]. In this study, most of the visible soiling on reprocessed devices occurred in areas visualized only after irreversible device disassembly and may be missed during a standard reprocessing protocol.

A handful of studies evaluating reprocessed SUDs have illustrated the retention of biological soils despite cleaning procedures. In a study that examined surgical scissors, forceps, rasps, and drill bits, complex device features were found to harbor protein, and biofilm encapsulated bacteria [23]. Stainless steel surgical instruments intended for multiple use have also proven to harbor organic material despite reprocessing procedures [24]. In addition to visible, tissue-colored stains, EDS studies of reprocessed ablation catheters and electrosurgical pencils revealed intact surface coatings of silicon and oxygen on new devices, while reprocessed devices harbored significantly higher amounts of carbon, sulfur, and sodium in the cell shaped residual material and degradation of surface coatings [25, 26]. This is consistent with observations made during this study, as illustrated in Table 2. In addition to the risk of microbial transmission, the presence of organic material on medical devices has been implicated in inflammatory reactions and septic responses due to the presence of endotoxins [27].

Protection of microorganisms against sterilization can occur by multiple mechanisms including occlusion in tightly bound biofilms or microbial masses [28–30]. Biofilms have been shown to take several forms, including amorphous precipitates comprised of sulfur, calcium, and phosphorus encapsulating bacterial cells along with high concentrations of carbon and oxygen [30]. In this study, atomic and morphological analyses of the remnant materials suggest that bacterial biofilms may be present on reprocessed VSDs. For future studies, a more extensive EDS analysis of films could be conducted to more thoroughly characterize the observed remnant material and compare it to known substances. Additionally, a study with a larger SEM sample including increased magnification could also be used to further characterize the morphology of the remnant material.

For the devices tested in this study, sterility testing resulted in unacceptable failure rates; however, further testing with reprocessed devices that have been heated to the estimated operating temperature within the culture media is advised. Due to different thermal expansions (polymer vs. metal), the effects of material expansion during device use may not be fully appreciated. When used during a procedure, it is possible that more remnant material may be exposed to patient fluids and tissues and subsequently increase the odds of disease transmission from the reprocessed device to the patient. A published study on the sterility of endoscopic forceps and snares found results similar to this study where 1 of 10 reprocessed devices failed bioburden testing (immersion in broth) [31]. They also found that when sterility testing was conducted in accordance with the United States Pharmacopeia standard, that 70% (14/20) of the devices showed growth of gram-positive bacteria at 14 days [31]. Together, this indicates that further work is needed to validate the sterility of reprocessed SUDs.

Hemoglobin testing had similar outcomes to other assays, finding a number of reprocessed devices positive. During testing, a majority of the observed soiling on reprocessed devices appeared inaccessible to the Luminol mixture, which may have inhibited the stain from binding consistently. There was evidence of a hydrophobic surface based on beading of the water-based Luminol solution. We hypothesize that the heat generated during device use and the reprocessing process may have bonded the biological material to the plastic jaw, which is supported by studies evaluating a series of stainless steel surgical instruments that had undergone several sterilization cycles [23, 24]. Future work is needed to confirm bonding and effectively solubilize film samples to enable identification and quantification of the remnant material.

In addition to biological contamination, reprocessed devices may also have structural damage compared to new devices. Inspection of the surface of the plastic jaws using SEM suggests degradation (in the form of cracking and pitting), perhaps from thermal cycling and/or exposure to harsh reagents. The diffuse presence of glass fibers indicates degradation of the polymer matrix. The mechanical integrity of reprocessed devices should be assessed for catastrophic brittle failure along with exploration of the effects of glass fibers escaping into tissues. Two studies assessed the mechanical performance degradation of LigaSure™ devices, as in this study, and discovered most devices failed due to inconsistent pressure application after 9 or 15 activation cycles, resulting in incomplete vessel sealing [32, 33]. Incomplete vessel sealing can go undetected until after completion of the surgery, resulting in internal hemorrhaging [29]. Combined, these observations suggest that there are physical defects in reprocessed devices, which introduces functionality questions beyond infection risk.

## Conclusions

In conclusion, this study provides evidence that VSDs have design features that can harbor significant amounts of organic material throughout reprocessing. With multiple assessments for the presence of biological material, 96% of reprocessed devices (81/84) experienced at least one failed test during this study. Given the well-established sterilization guidelines that mandate a device be clean for sterilization to be effective, sterilization procedures may not be effective for reprocessed VSDs. To that end, evidence from reprocessed VSDs found a sterility level ≤ 6^–3^ that exceeded the recommended limit of 10^–6^ by 3 orders of magnitude. Nosocomial infections carry serious health and economic consequences, such as infection, sepsis, wound healing problems, re-operation, and extended hospital stays that generate significant financial burdens and increases in medical waste generation making the balance of the risks, such as insufficient sterilization and material/performance degradation, and benefits of reprocessing single-use devices difficult.

The results of this study suggest that reprocessing procedures for SUDs should be re-examined with respect to ensuring cleanliness prior to sterilization. In alignment with the position of the Society of Gastroenterology Nurses and Associates, it is the opinion of the authors that critical medical devices designed for single use, such as VSDs, should not be re-used. Regulatory tracking and reporting systems are not conducive to obtaining reliable data comparing adverse events between new and reprocessed SUDs, which makes it difficult to assess clinical implications of remnant material on reprocessed medical devices [34]. This was supported by our experience which showed the external packaging of the devices were different; however, the reprocessed devices retained the original manufacturer labeling. Accurately discerning differences in infection rates between new and reprocessed devices would require information about device history (obtained from the reprocessing company) and operating room records containing specific manufacturer and lot information for all devices used. Additional clinical research is needed to examine the potential link between reprocessed SUDs and surgical site infections or other adverse events.
